# Linkages between oral commensal bacteria and atherosclerotic plaques in coronary artery disease patients

**DOI:** 10.1038/s41522-016-0009-7

**Published:** 2016-12-19

**Authors:** Jyoti Chhibber-Goel, Varsha Singhal, Debaleena Bhowmik, Rahul Vivek, Neeraj Parakh, Balram Bhargava, Amit Sharma

**Affiliations:** 1 Molecular Medicine Group, International Centre for Genetic Engineering and Biotechnology, New Delhi, India; 2 Cardiothoracic Sciences Centre, All India Institute of Medical Sciences, New Delhi, India

## Abstract

Coronary artery disease is an inflammatory disorder characterized by narrowing of coronary arteries due to atherosclerotic plaque formation. To date, the accumulated epidemiological evidence supports an association between oral bacterial diseases and coronary artery disease, but has failed to prove a causal link between the two. Due to the recent surge in microbial identification and analyses techniques, a number of bacteria have been independently found in atherosclerotic plaque samples from coronary artery disease patients. In this study, we present meta-analysis from published studies that have independently investigated the presence of bacteria within atherosclerotic plaque samples in coronary artery disease patients. Data were collated from 63 studies covering 1791 patients spread over a decade. Our analysis confirms the presence of 23 oral commensal bacteria, either individually or in co-existence, within atherosclerotic plaques in patients undergoing carotid endarterectomy, catheter-based atherectomy, or similar procedures. Of these 23 bacteria, 5 (*Campylobacter rectus*, *Porphyromonas gingivalis*, *Porphyromonas endodontalis*, *Prevotella intermedia*, *Prevotella nigrescens*) are unique to coronary plaques, while the other 18 are additionally present in non-cardiac organs, and associate with over 30 non-cardiac disorders. We have cataloged the wide spectrum of proteins secreted by above atherosclerotic plaque-associated bacteria, and discuss their possible roles during microbial migration via the bloodstream. We also highlight the prevalence of specific poly-microbial communities within atherosclerotic plaques. This work provides a resource whose immediate implication is the necessity to systematically catalog landscapes of atherosclerotic plaque-associated oral commensal bacteria in human patient populations.

## Heart disease: Links to oral bacteria

A review of bacterial populations in the mouth and in diseased arteries will help research into the role of bacteria in heart disease. Amit Sharma and colleagues at the International Centre for Genetic Engineering and Biotechnology, with co-workers at the All India Institute of Medical Sciences, both in New Delhi, India, analyzed 63 studies covering 1791 patients spread over a decade. They summarize evidence of 23 types of oral bacteria that are also found in atherosclerotic plaques in artery walls. The review also cataloged the proteins secreted by the bacteria and discussed possible involvement of these proteins in the migration of bacteria through the bloodstream. Full genetic details are available for 19 of the 23 bacterial species, which should greatly assist further investigations into the significance of bacteria in the onset of heart disease.

## Background

Human microbiome is now recognized as a central player in human health, and the oral microbiome is of increasing significance in human biology. The oral cavity includes several microbial habitats and is the central channel for entry of bacteria into the human body. It constitutes the second most complex ecological system in human body after the gut microbiome, with over 700 species of bacteria and >5 billion bacteria.^1^ These gram-positive or gram-negative bacteria colonize periodontal surfaces and are part of the saliva. Further, depending on oxygenation conditions, they may be aerobic (obligates or facultative) or anaerobic (obligates or facultative). In healthy conditions, the oral bacterial community of ~700 species is stable but remains susceptible to alteration in its population structures due to infections or other stresses (Human Oral Microbiome Database). Increasing evidence on known molecular diversity of bacteria and recent advances in culture-independent techniques has validated the involvement of oral microbiome in several autoimmune and metabolic events such as obesity, diabetes and cardiovascular diseases.^2–5^

In 2012, an estimated 17.5 million people died from cardiovascular diseases, of which 7.4 million deaths were due to coronary artery disease (CAD) arising from atherosclerosis.^6^ CAD is a chronic inflammatory disorder characterized by narrowing of the coronary artery due to plaque formation, and results in blocking or reducing oxygen-rich blood supply to the heart that may subsequently cause myocardial infarction.^7^ The major content of an atherosclerotic plaque is atheroma that is composed of macrophages, cholesterol, smooth muscles and dystrophic calcification.^8^ Interestingly, with the development of targeted microbial techniques a number of oral bacteria have been identified in atherosclerotic plaque samples.

The link between dental disease and CAD was first established ~23 years ago when De Stefano et al. reported an increased risk of atherosclerotic plaque formation in a group of patients with periodontitis (25 % higher) based on 14 years of research on 9760 individuals aged between 25–74 years.^9^ More recent studies have correlated oral microbial dysbiosis/infections with obesity as well as diabetes, two known drivers of CAD.^5^ The oral route is a key avenue for entry of bacteria into the human body, and the prevailing hypothesis for the above-established link suggests that there is a flow of bacterial toxins and/or bacterial components into the bloodstream, leading to exaggerated release of inflammatory mediators that can drive CAD.^10^ For example, it has been demonstrated that lipopolysaccharide (LPS)-derived products released during endotoxemia are contributors in the host–bacteriadialog, whereas LPS increases serum cholesterol levels by increasing low-density lipoprotein (LDL) thereby increasing the risk of developing CAD.^11^ Similarly, other bacteria-derived components such as DNA or membrane phospholipids from oral cavity may also reach the blood stream, and finally into adipose and vascular tissues. Thus, the oral cavity may represent a significant source of bacterial mediators (direct or indirect) that may have an impact on CAD.

**Fig. 1:**
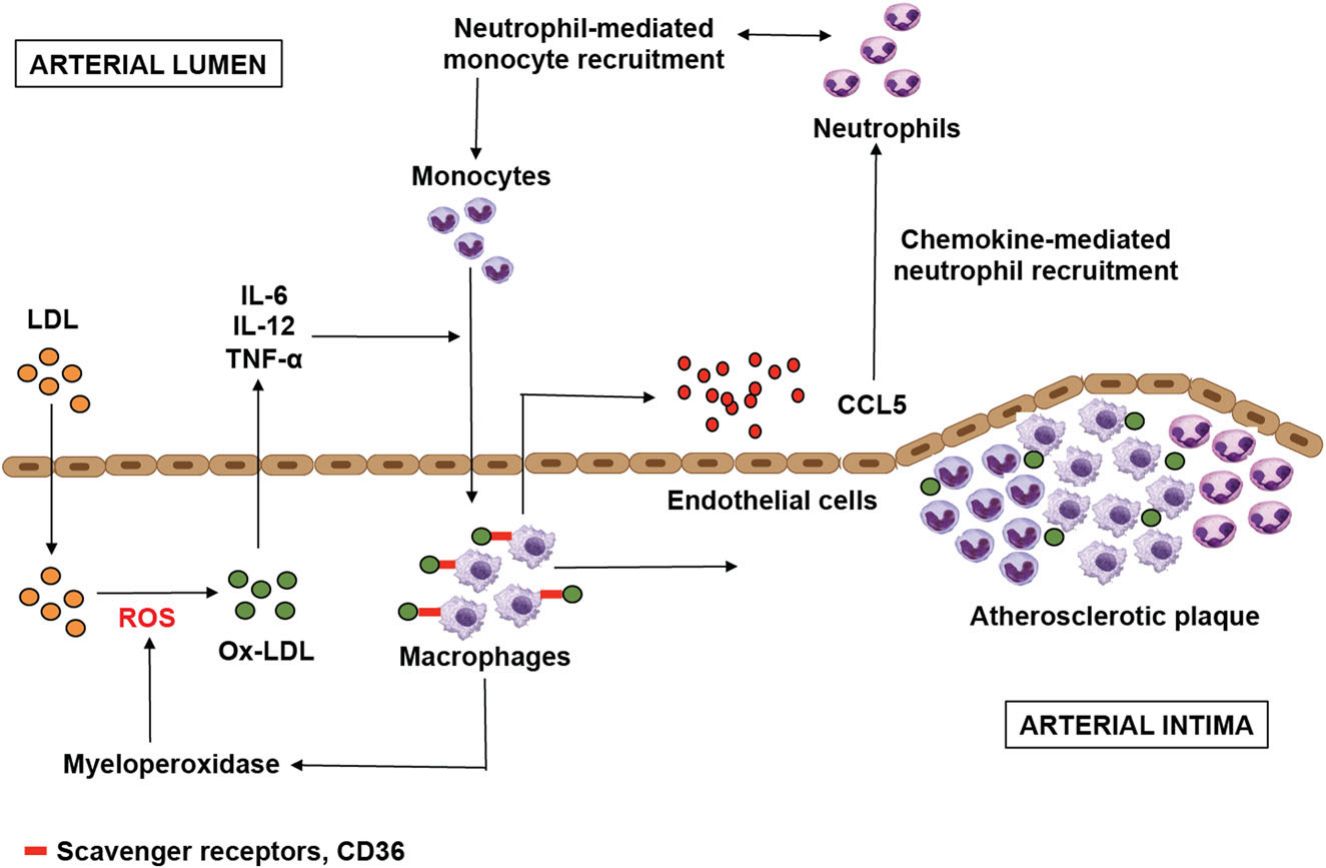
Schematic representation of inflammatory mechanisms involved in pathogenesis of atherosclerosis and plaque formation. LDL is retained in arterial intima via ionic interactions with endothelial cells, leading to the enzymatic oxidative modification of LDL into OxLDL. This is followed by secretion of pro-inflammatory cytokines that leads to the differentiation of monocytes into macrophages. Macrophages secrete more chemokines and mediate recruitment of neutrophils via scavenger receptors and further attract monocytes. Macrophages retained in arterial intima get converted into foam cells leading to the formation of atherosclerotic plaques

In order to assess the role of oral bacteria as inflammatory stimuli, it is important to understand the contribution of human immune system toward the formation of atherosclerotic plaque. The process of plaque formation in coronary arteries is initiated by the accumulation of LDL across the endothelium, leading to its retention in intima.^12, 13^ Following this deposition, LDL undergoes oxidative modification via enzymes secreted by endothelial cells.^14^ Oxidized LDL (OxLDL) trapped in the intima stimulates endothelial cells to secrete pro-inflammatory molecules, chemotactic proteins and growth factors that help in the recruitment of monocytes to the arterial wall and promote their differentiation and proliferation to macrophages.^15^ Macrophages then adhere to the endothelium and phagocytose OxLDL, leading to the formation of foam cells. The continued damage to endothelial cells leads to secretion of growth-stimulating factors, which in turn stimulates the proliferation of macrophages to function as foam cells.^16^ These foam cells then rupture and accumulate on the arterial wall. Following the deposition of lipids within the arterial wall, immune cells such as monocytes, leukocytes, B-cells, T-cells, neutrophils, dendritic cells (DC) and mast cells are recruited, which is the hallmark of atherosclerotic plaque formation^17–22^ (Fig. 1).

Atherosclerotic plaque formation may also be initiated by oral microbial dysbiosis/infection resulting in an inflammatory stimulus. For example (1) chronic periodontitis, a well-studied oral microbial disease with immunological implications, begins as an inflammation localized to the soft tissues (gingivitis) caused by resident biofilm that forms on tooth surfaces at the gingival margin. If left untreated, this leads to damage of connective tissue, periodontal ligament and bone.^23, 24^ (2) Gingival ulceration in periodontitis results in bacteraemia and can provide an additional inflammatory stimulus for atherosclerotic plaque formation.^25, 26^ The inflammatory cytokines produced and additional chemotactic agents lead to changes in the endothelium, e.g. via upregulation of adhesion molecules. These changes promote interactions with leukocytes, further promoting their migration into the intimal layer of the artery. Activation of the endothelium also results in the release of chemotactic cytokines, further attracting monocytes or other cells that form a vicious cycle leading to plaque formation.^8^

The aims of our study were to (i) collate and analyze associations between atherosclerotic plaque-associated bacteria in CAD patients from numerous independent studies, (ii) determine non-cardiac distribution of the oral bacteria in human body, (iii) dissect probable entry routes of oral bacteria into the coronary vasculature, (iv) highlight the plethora of proteins and peptides that are secreted by these bacteria, and finally (v) analyze the establishment of poly-bacterial communities within the plaques (Table 1).

**Table 1:** Techniques used for identification of the 23 atherosclerotic plaque-associated bacteria

Atherosclerotic plaque-associated bacteria	Detection platform	Percentage of bacteria present in atherosclerotic plaque samples	Reference
*Aggregatibacter actinomycetecomitans*	PCR	71.4 % [5/7]	^76^
[Phylum: Proteobacteria]	16S rRNA	66.67 % [28/42]	^77, 78^
	mAb	17 % [5/29]	^25, 79^
	16S rRNA	21.87 % [7/32]	^80^
	16S rRNA	18 % [9/50]	^81^
	16S rRNA	25.9 % [7/27]	^82^
	RT-PCR	46.2 % [18/39]	^83, 84^
	16S rRNA	29.4 % [15/51]	^85, 86^
*C*hlamydiae *pneumoniae*	mAb	20.6 % [6/29]	^25, 79^
[Phylum: Chlamydiae]	16s rRNA	35.4 % [11/31]	^25, 80^
	16s rDNA	18 % [9/50]	^25, 81^
	ICC/PCR	48 % [11/23]	^87^
	16S rRNA	51.5 % [17/33]	^88^
	MIF IgA	32.6 % [63/193]	^89^
	MIF IgG	61.7 % [119/193]	^89^
	16S rRNA	26 % [12/46]	^90^
	PCR	42 % [102/241 sections (10 samples)]	^91^
	PCR	69 % [11/16]	^92^
	Immunofluorescence	79 % [71/90]	^93^
	PCR	70 % [42/60]	^94^
	IgG antibody	61.7 % [50/81]	^95^
*Campylobacter rectus*	16S rRNA	9.52 % [4/42]	^77, 78, 96^
[Phylum: Proteobacteria]	PCR	11.7 % [6/51]	^83, 85^
	16S rRNA	21.51 % [11/51]	^83, 85, 97^
	16S rRNA	15.7 % [8/51]	^98^
	16S rRNA	21.51 % [11/51]	^82^
*Enteroacter hormaechei*	16S rRNA	50 % [134/268]	^4^
[Phylum: Proteobacteria]	16S rRNA	40 % [2/5]	^99^
*Eikenella corrodens*	16S rRNA	54.76 % [23/42]	^77, 78^
[Phylum: Proteobacteria]	PCR	15.6 % [8/51]	^96^
	16S rRNA	27.45 % [14/51]	^98^
*Fusobacterium nucleatum*	16S rRNA	50 % [21/42]	^77, 78^
[Phylum: Fusobacteria]	Monoclonal antibody	34 % [10/29]	^79, 25^
	PCR	21 % [4/19]	^100^
*Fusobacterium necrophorum*	–	–	^101–103^
[Phylum: Fusobacteria]			
*Helicobacter pylori*	IgA	55.4 % [107/193]	^89^
[Phylum: Proteobacteria]	IgM	44.6 % [86/193]	^89^
	16S rRNA	37 % [17/46]	^90^
	IHC	57.8 % [22/38]	^104^
	PCR	92.16 % [47/51]	^105^
	IgG	67.9 % [55/81]	^95^
*Mycoplasma pneumoniae*	Seropositivity	14 % [396]	^106^
[Phylum: Tenericutes]	–	–	^107^
*Porphyromonas endodontalis*	–	–	^108^
[Phylum: Bacteriodetes]			
*Porphyromonas gingivalis*	16S rRNA	78.57 % [33/42]	^77, 78^
[Phylum: Bacteriodetes]	PCR	71.43 % [5/7]	^76^
	16S rRNA	67 % [134]	^4^
	mAb	52 % [15/29]	^79, 25^
	16S rRNA	22.27 % [6/22]	^25, 80^
	16S rRNA	26 % [13/50]	^81, 25^
	PCR	47.4 % [9/19]	^100^
	PCR	51 % [27/53]	^109, 110^
	PCR	43.1 % [22/51]	^96^
	16S rRNA	45.1 % [23/51]	^83, 85^
	16S rRNA	21.6 % [11/51]	^83, 85, 97^
	RT-PCR	53.8 % [21/39]	^83, 84^
	16S rRNA	45.1 % [23/51]	^98^
	16S rRNA	7.4 % [2/27]	^82^
*Prevotella intermedia*	mAb	41 % [12/29]	^25, 79^
[Phylum: Bacteriodetes]	16S rRNA	9.37 % [3/32]	^25, 80^
	16S rRNA	14 % [7/50]	^25, 81^
	PCR	21 % [4/19]	^100^
	PCR	15 % [8/53]	^23, 110^
	PCR	19.6 % [10/51]	^96^
	RT-PCR	79.3 % [23/29]	^83, 84^
	PCR	71.43 % [5/7]	^76^
	16S rRNA	3.7 % [1/27]	^82^
*Prevotella nigrescens*	PCR	15.6 % [8/51]	^96^
[Phylum: Bacteriodetes]	RT-PCR	17.9 % [7/39]	^83, 84^
*Pseudomonas aeruginosa*	16S rRNA	40 % [6/15]	^74^
[Phylum: Proteobacteria]			
*Pseudomonas lut*eola	16S rRNA	100 % [15/15]	^111^
[Phylum: Proteobacteria]			
*Streptococcus gordonii*	16S rRNA	19.4 % [-]	^82^
*Streptococcus mitis*	16S rRNA	19.4 % [-]	
*Streptococcus mutans*	16S rRNA	74.1 % [20/27]	
*Streptococcus oralis*	16S rRNA	3.7 % [1/27]	
*Streptococcus sanguinis*	16S rRNA	25.9 % [7/27]	
[Phylum: Firmicutes]			
*Treponema denticola*	PCR	43 % [23/53]	^83, 109^
[Phylum: Spirochaetes]	16S rRNA	44.4 % [12/27]	^82^
	PCR	35.2 % [18/51]	^96^
	16S rRNA	49.01 % [25/51]	^83, 85^
	16S rRNA	27.4 % [14/51]	^83, 85, 86^
	16S rRNA	23.1 % [6/26]	^82, 86^
	16S rRNA	49.01 % [25/51]	^98^
*Tannerella forsythia*	16S rRNA	61.9 % [26/42]	^77^
[Phylum: Bacteriodetes]	PCR	100 % [7/7]	^76^
	mAb	34 % [10/29]	^25, 79^
	16S rRNA	30 % [15/50]	^25, 81^
	PCR	10.5 % [2/19]	^100^
	PCR	19.6 % [10/51]	^96^
	16S rRNA	5.9 % [3/51]	^78, 83, 85^
	RT-PCR	25.6 % [10/39]	^83, 84^
*Veillonella*	16S rRNA	10 % [2/20]	^112^
[Phylum: Firmicutes]	16S rRNA	100 % [13/13]	^111^

## Results

### Distribution of oral bacteria in atherosclerotic plaques

**Fig. 2:**
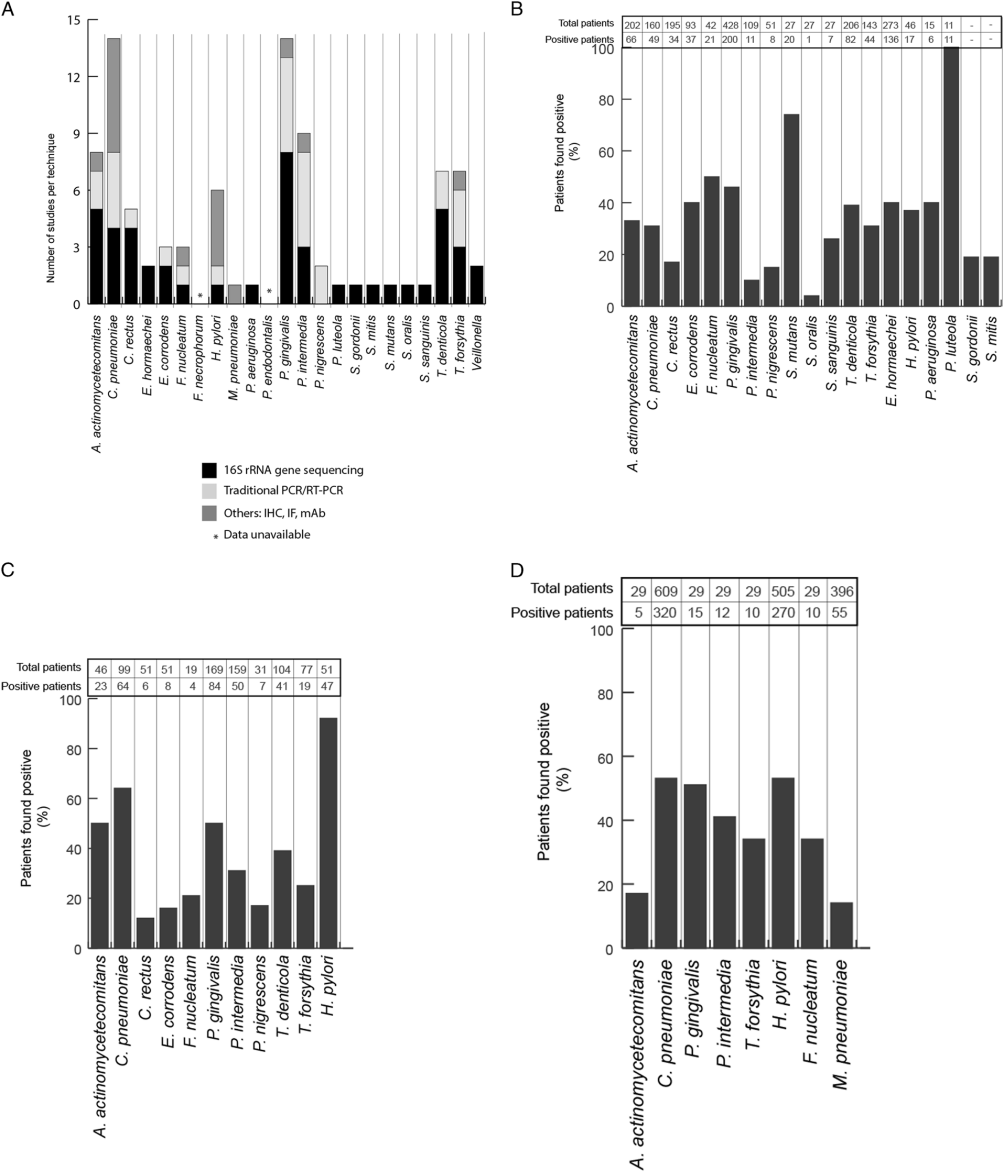
**a** Graphical representation of the techniques used to identify the atherosclerotic plaque-associated bacteria in CAD patients. *Y*-axis represents the number of studies reporting the presence of bacteria in the plaque samples, while *X*-axis depicts the corresponding bacteria. The cohort of 23 commensal bacteria is dominated by gram-negative bacteria with the exception of *Streptococcus sp.*, which are gram-positive. Of these 23 atherosclerotic plaque-associated bacteria, *A. actinomycetemcomitans*, *C. rectus*, *E. corrodens*, *E. hormaechei*, *S. gordonii*, *S. mitis*, *S. mutans*, *S. oralis*, *S. sanguinis*, *H. pylori*, and *P. aeruginosa* are facultative anaerobes, while *C. pneumoniae*, *F. necrophorum*, *F. nucleatum*, *M. pneumoniae*, *P. endodontalis*, *P. gingivalis*, *P. intermedia*, *P. nigrescens*, *T. denticola*, *and T. forsythia* are obligatory anaerobes. There are two exceptions in *P. luteola* (aerobe) and *Veillonella* (anaerobe). *IF* immunofluorescence, *IHC* immunohistochemistry, *mAb* monoclonal antibodies. **b** Atherosclerotic plaque-associated bacteria identified using 16S rRNA technique. *X*-axis represents the % of patients positive for the bacteria identified using 16S rRNA gene sequencing, while *Y*-axis represents the corresponding bacteria. Total number of study subjects vs. positive patients is mentioned on the *top* of graph. **c** Atherosclerotic plaque-associated bacteria identified using traditional PCR techniques. *X*-axis represents the % of patients positive for the bacteria identified using traditional PCR techniques, while *Y*-axis represents the corresponding bacteria. Total number of study subjects vs. positive patients is mentioned on the *top* of graph. **d** Atherosclerotic plaque-associated bacteria identified using immunofluorescence, immuno-histochemistry, and antibody screening methods. *X*-axis represents the % of patients positive for the bacteria identified using multiple techniques, while *Y*-axis represents the corresponding bacteria. Total number of study subjects vs. positive patients is mentioned on the *top* of graph

Our literature search resulted in the selection of 63 studies and the identification of 23 bacteria that individually or otherwise co-existed in the studied human atherosclerotic plaque samples. Techniques used for the identification of bacteria within the atherosclerotic plaque samples included traditional methods like (1) categorizing on the basis of morphological characteristics, (2) culturing and isolation of colonies from samples. In addition, more modern techniques like immunohistochemistry, immunofluorescence, real-time polymerase chain reaction (PCR), nested PCR, and 16S rRNA gene sequencing were also used to identify bacteria. Among these techniques, 16S rRNA gene sequencing is the most reliable, cost effective, and scalable method when studying a large group of samples (Fig. 2). Our analyses show that 16S rRNA gene sequencing was the dominant technique (used 48 times) over traditional PCR methods (29 times) and other techniques like immunofluorescence and immune-histochemistry (15 times). Specifically, only 16S rRNA gene sequencing methodology identified *Streptococcus sp.*, *Enterobacter hormaechei*, *Pseudomonas aeruginosa*, *Pseudomonas luteola*, *Veillonella* (Fig. 2a), and overall it identified 16 of the 23 atherosclerotic plaque-associated bacteria in CAD patients (Figs. 2b–d).

### Atherosclerotic plaque-associated bacteria in non-cardiac organs

**Fig. 3:**
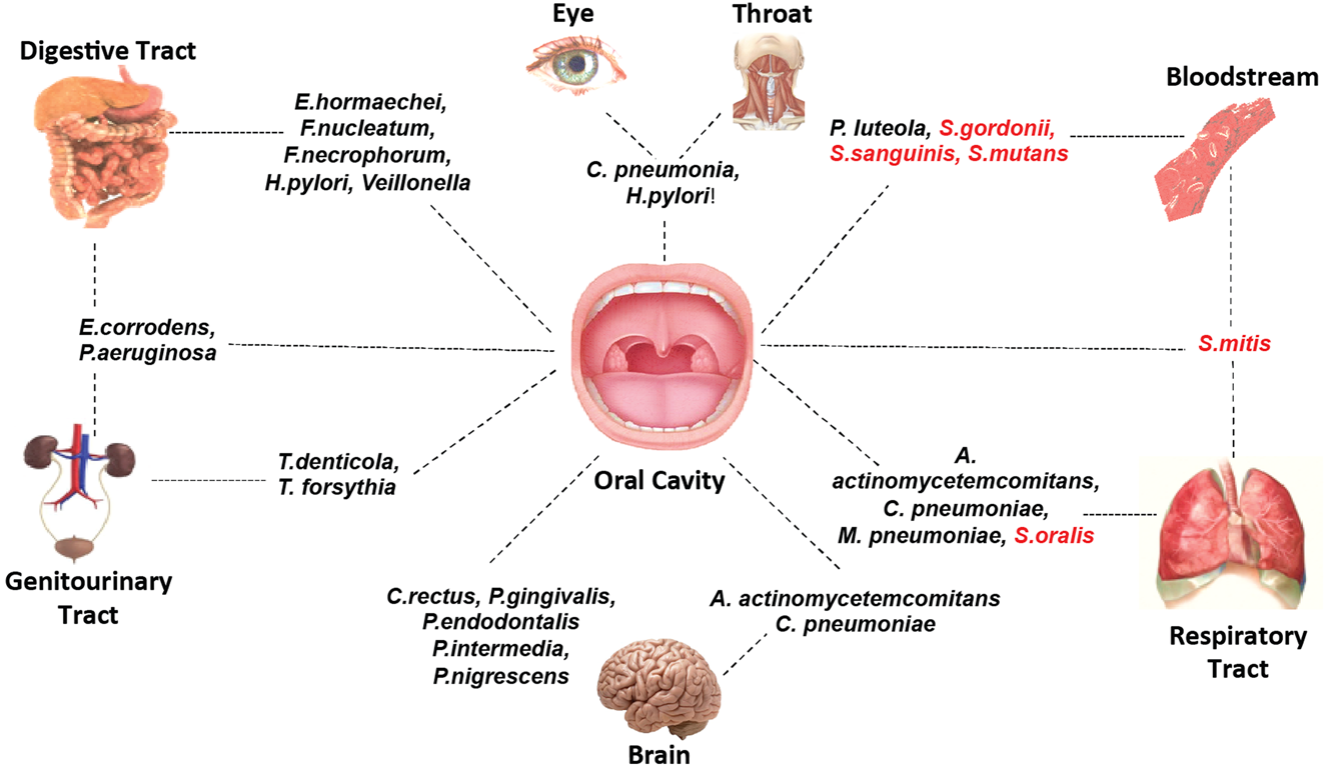
The tissue localization of the 23 oral commensal bacteria associated with atherosclerotic plaque samples from CAD patients. Sixteen of the 23 atherosclerotic plaque-associated bacteria were not unique to atherosclerotic plaque samples and are present in multiple non-cardiac organs (gram-negative microbes are in *red*)

**Fig. 4:**
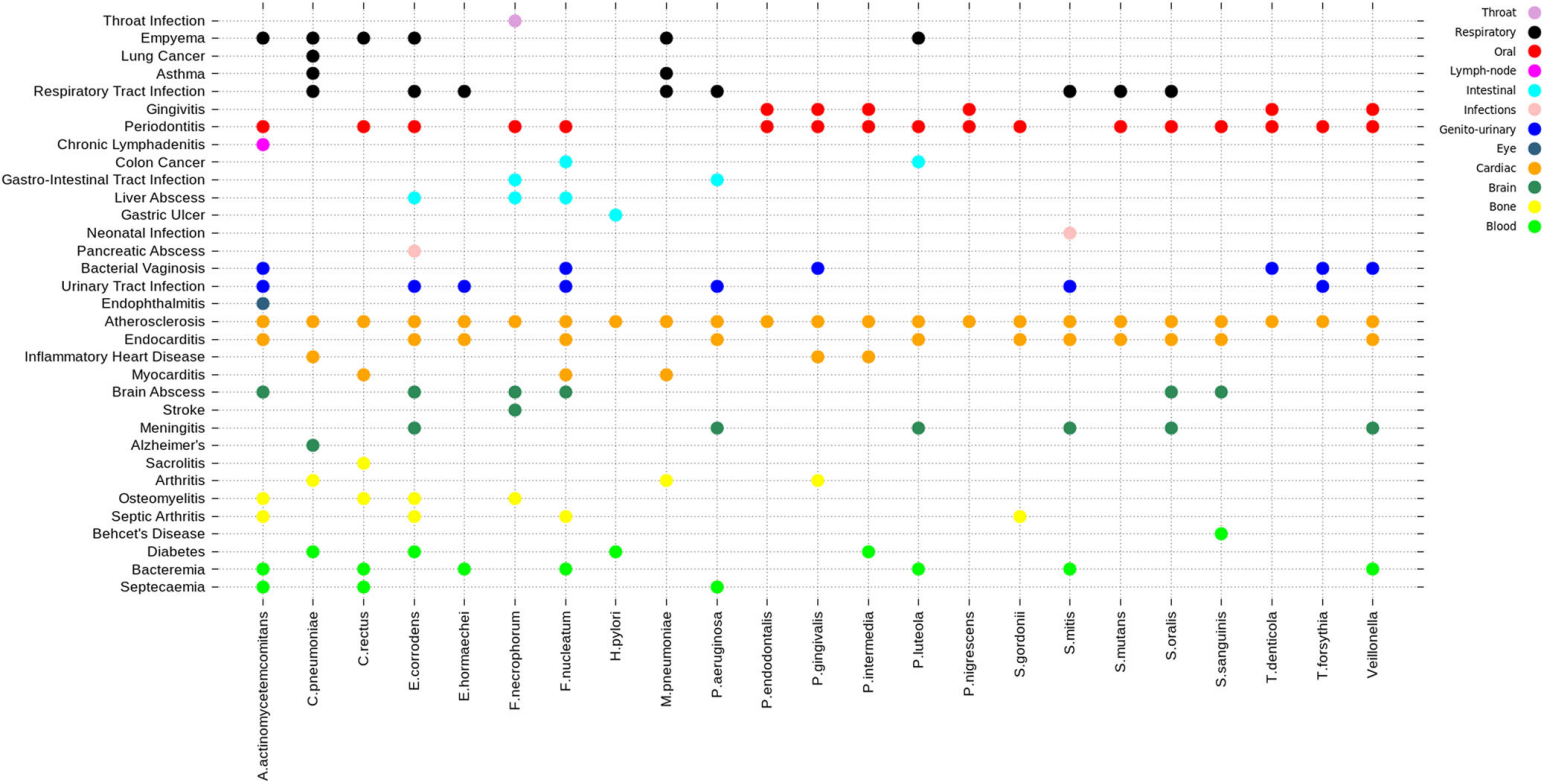
Multiple diseases caused by the atherosclerotic plaque-associated bacteria. Dot plot graph for cardiac and non-cardiac diseases caused by the atherosclerotic plaque-associated oral bacteria divided into categories based on their tissue localization (prepared using GG plot)

On investigating the tissue distribution of 23 atherosclerotic plaque-associated bacteria, we found that some of these were found in several body organs (Fig. 3). The translocation of these bacteria into the bloodstream and subsequently to multiple organs may be triggered by tissue damage via periodontal probing, scaling, and tooth extractions, and/or aided by the proteins secreted by these bacteria. For example, *Chlamydiae pneumoniae* can lead to chronic obstructive pulmonary disease,^27^ sexually acquired reactive arthritis,^28^ asthma,^29^ increase the risk of developing lung cancer, and is present within brain regions of Alzheimer patients.^30^ In addition, *C. pneumoniae* reinfection accelerates the development of insulin resistance and diabetes in obese C57BL/6 mice.^31^ Similarly, *Aggregatibacter actinomycetemcomitans* is responsible for brain abscess, infectious arthritis,^32^ rib destruction^33^ as well as infective endocarditis.^34^ Of the 23 bacteria, *Fusobacterium nucleatum* is associated with inflammatory bowel disease,^35^ ulcerative colitis,^36^ and intestinal tumorigenesis.^37, 38^ The most studied of these are *P. gingivalis* and *Helicobacter pylori* and their detailed associations are depicted and categorized on the basis of tissue or organ system affected (Fig. 4). These data together highlight the complex associative underpinnings of oral commensals and human body organs.

### Proteins/peptides predicted to be secreted by atherosclerotic plaque-associated bacteria

**Fig. 5:**
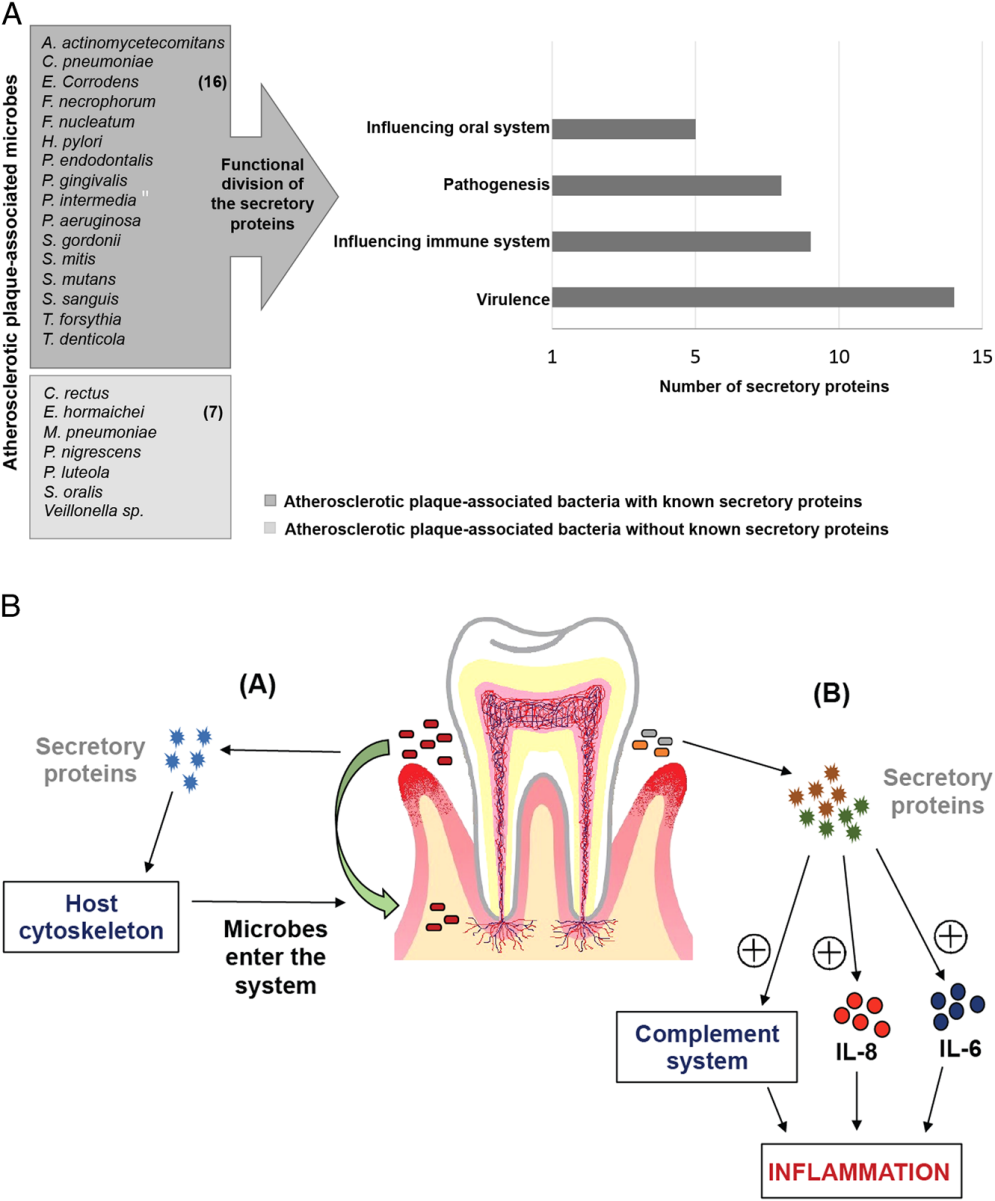
Proteins secreted by the atherosclerotic plaque-associated bacteria. **a** Histogram representing the number of secretory protein/peptides and proteases from atherosclerotic plaque-associated bacteria. **b** The gingival crevice is a habitat to many oral microbes that secrete proteins, peptides and proteases. (1) Secretory peptides and proteases are likely responsible for altering the host actin cytoskeleton in the gingival epithelium leading to microbial entry into the system. (2) These secreted proteins can also activate the immune system causing inflammation. Primarily, cytokine-mediated (IL-6 and IL-8) inflammation is associated with atherosclerotic plaque formation. Certain proteases cause inflammatory response by activating the complement system

We cataloged 36 predicted secretory proteins from 16 plaque-associated bacteria (Table 2). These proteins likely have multiple functions that include aiding in bacterial pathogenesis, increasing the virulence of the bacteria, and/or regulating host immune responses. Further, we analyzed these 36 secretory proteins in context of the host immune system and categorized them on the basis of their potential to influence oral cavity and immune system that could lead to inflammation (Fig. 5a). Both *H. pylori* and *P. gingivalis* have been studied extensively in context of their involvement in various disease pathologies. Secretory proteins such as gingipains from *P. gingivalis* and Hpn from *H. pylori* are known to activate cytokine secretion (mainly IL-6 and IL-8).^39–41^ Similarly, the CD-14 binding protein of *S. sanguinis* also results in secretion of host cytokines IL-6 and IL-8.^42^ Further, the protein SerbB secreted by *P. gingivalis* modulates host cytoskeleton, thus aiding microbes to enter host tissues.^43^ The leukotoxin (Lkt A) secreted by *Fusobacterium necrophorum*,^44^ Dup A,^45^ HtrA^46^ of *H*. *pylori*, and interpain A of *P. intermedia* ^47^ all serve as virulence factors for their respective bacteria, assisting them in infecting the host cells. The elastase A (Las A) and elastase B (Las B) of *P. aeruginosa* also have probable roles in bacterial pathogenesis.^48^

**Table 2:** Proteins and peptides secreted by atherosclerotic plaque-associated bacteria and their potential roles in disease aetiology

Microbes	Secreted proteins	Mol wt. (kDa)	Function	Reference
*A. actinomycetemcomitans*	Leukotoxin (LtxA)	114	Targets leukocyte function antigen-1 on activated WBC triggering lysosomal-mediated cell death.	^113^
	Cytolethal distending toxin (Cdt)	31.5	Inhibits macrophage phagocytosis and subverts cytokine production	^114^
*C. pneumoniae*	(CPAF) chlamydial protease-like or proteasome-like activity factor	70	Disrupts host MHC antigen presentation	^115, 116^
*E. corrodens*	Corrodecin (bacteriocin)	23.6	Potential role at the periodontal site	^117^
	Hydrolytic enzymes (includes proline aminopeptidase, thiol-dependent haemolysin and esterase activities)	–	Proposed to act against proline residues in collagen, immunoglobulin and complement proteins	^118^
*F. necrophorum*	Leukotoxin (LktA)	335.9	Virulence factor	^49^
*F. nucleatum*	Fusolysin	115	–	^119^
*H. pylori*	CagA oncoprotein	132.4	Virulence factor. Reprograms gastric epithelial cells	^120^
	VacA exotoxin (Vacuolating cytotoxin A)	88	Virulence factor	^121^
	HP-NAP (neutrophil activating protein)	204	Activates innate immunity	^122^
	CagL Y58/E59 (amino acid polymorphisms)	26.8	Increases hypochlorhydria; disrupts cell membrane integrity	^123^
	Hpn	7	Modulates cytokine secretion	^46^
	Tip-α	19.6	Bacterial pathogenesis	^124^
*H. pylori*	HP0175	34	Virulence factor	^125^
	HcpE(HP0235)	39.4	Virulence factor	^126^
	DupA (Duodenal ulcer producing)	20	Virulence factor	^50^
	HtrA (high temperature requirement—A) chaperones and serine protease	48	Virulence factor	^51^
*P. endodontalis*	35,406 protease	88	Role in pathogenesis and nutrition of the microbe	^127^
*P. gingivalis*	Arginine gingipain	81	Regulates IL-8; modulates microbiome population	^44, 45^
	Lysine gingipain	60		
	SerB protein	45.9	Entry and survival of *P. gingivalis* in the epithelial cells	^48^
	Fimbrilin A	43	Hemagglutinating activity	^128^
*P. intermedia*	Interpain A	27	Potential virulence factor	^52^
*P. aeruginosa*	Alkaline protease	50	Suggested role in pathogenesis	^53^
	(AprA)	45.5		
	Elastase A (LasA)	53.7		
	Elastase B (LasB)	26		
	Protease IV			
	LepA (large exoprotease)	66.3	Activation of pro-inflammatory pathway	
*S. gordonii*	A soluble GAPDH (glyceraldehyde 3-phosphate dehydrogenase)	35.9	Virulence factor	^129^
*S. mitis*	Mitilysin (cholesterol-dependant cytolysin)	53	Cholesterol-dependent cytolysin	^130^
*S. mutans*	CSP (competence-stimulating peptide)	5.2	Inhibits *Candida albicans* morphological switch, found in oral cavity	^131^
*S. sanguinis*	FruA (exo-beta-D-fructosidase)	140	Multifunctional enzyme	^132^
	CD-14 binding protein	190	Stimulate cytokine synthesis	^47^
*T. forsythia*	Karilysin	52	Virulence factor	^133^
	KLIKK protease	–	Host protein degradation and pathogenicity	^134^
*T. denticola*	Dentipain (IdeT) cysteine protease	43	Virulence factor	^135^

We present a model for possible access routes of bacteria into the epithelial tissues (Fig. 5b). When in blood, commensal bacteria can invade the endothelial layer of the blood vessels with help of secretory proteins, and stimulate the production of pro-inflammatory cytokines such as monocyte chemo-attractant protein 1, IL-6, and IL-8.^49, 50^ These inflammatory cytokines can result in recruitment of DC, which then phagocytose oral bacteria and carry them through the blood stream until they are deposited in the vascular sites.^51^ This thus provides a potential entry point for the oral bacteria, and enables their migration from oral cavity into the blood stream—and feasibly to the coronary arterial system. In addition to the above, several proteins secreted by oral bacteria (Table 2) are capable of degrading oral mucosal membranes and periodontal pockets, again facilitating the entry of bacteria into the blood stream. It remains unclear whether oral bacteria nucleate atherosclerotic plaque formation or that they are deposited in the plaque site once it has developed.

### Poly-microbial community in atherosclerotic plaques

**Fig. 6:**
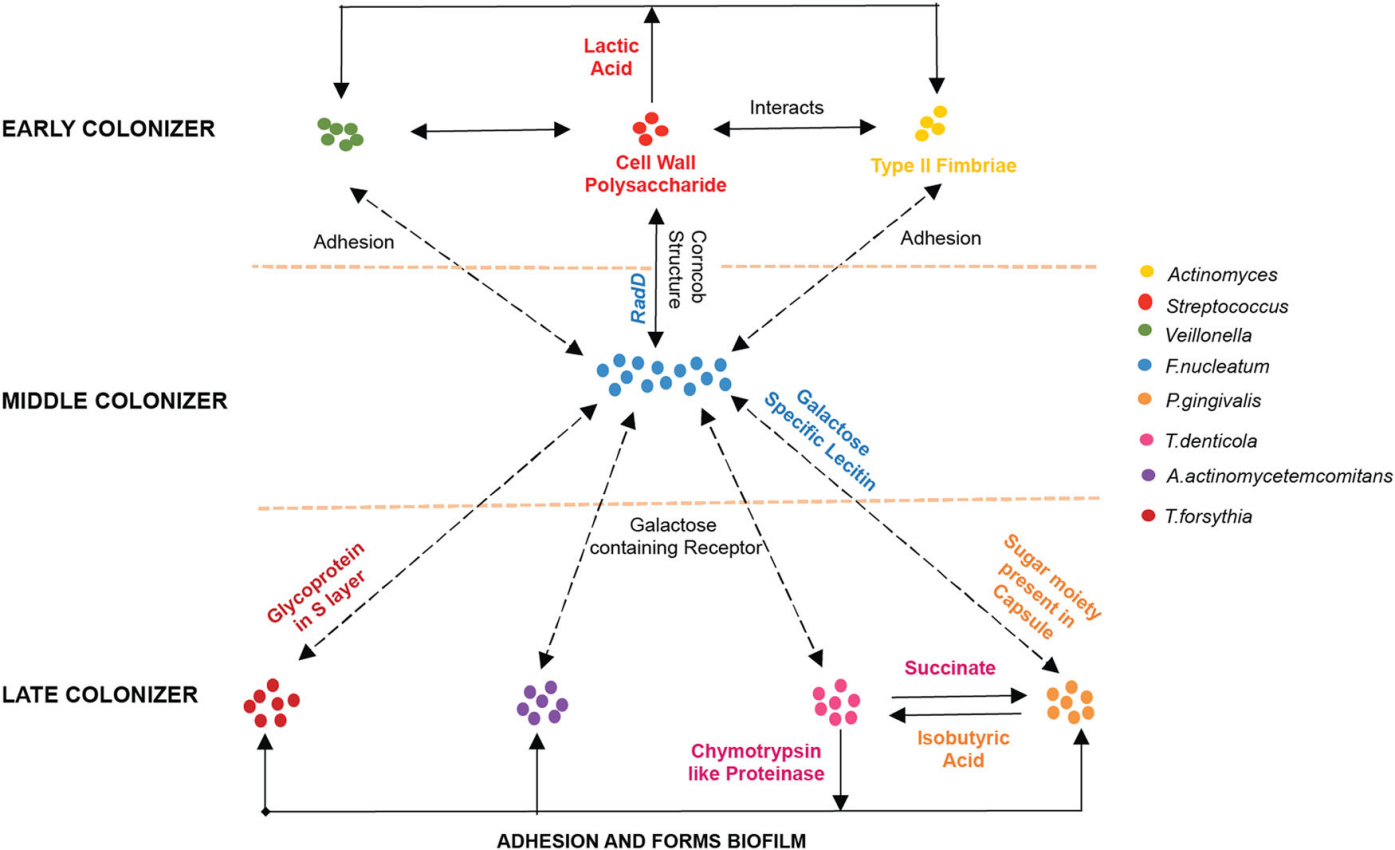
Atherosclerotic plaque-associated bacteria form biofilm structures within the atherosclerotic plaque samples. During initial phase of biofilm formation, early colonizers—*Veillonella*, *Streptococcus*, and *Actinomyces*—interact to establish an initial microenvironment supporting each other with the help of metabolic products. These bacteria act as a platform for the middle colonizer *F. nucleatum*, which then completes the biofilm formation by providing an adhering platform for the late colonizers—*T. forsythia*, *A. actinomycetemcomitans*, *T. denticola*, and *P. gingivalis*

The survival strategies adopted by commensal bacteria are of interest as they allow formation of a poly-microbial environment.^52^ With the exception of aerobe *P*. *luteola*, the other 22 atherosclerotic plaque-associated bacteria are either facultative or obligatory anaerobes. Under aerobic conditions, anaerobic bacteria have been shown to form biofilm structures, thus establishing an intricate and genetically varied microenvironment to survive.^53^ The process of biofilm formation in the oral cavity is initiated with the aggregation of early colonizers like *Actinomycetes*, *Streptococcus* and *Veillonella*. The *Actinomycetes* and *Streptococcus sp.* are present in almost equal ratio during the initial stages of biofilm formation in oral cavity.^54–56^ The *Streptococcus* and *Actinomyces* interact such that cell wall polysaccharide of *Streptococcus* binds with Type II fimbriae of *Actinomyces* resulting in first step towards biofilm formation.^57^ Further, the metabolic products of *Streptococcus sp.* such as lactic and pyruvic acid are exploited by *Actinomycetes* and *Veillonella* to support their own growth. Contrary to this, *Streptococcus sp.* convert excess lactic acid to hydrogen peroxide, thus preventing the attachment and growth of other periodontal pathogens.^58^ In the next phase of biofilm formation, *F. nucleatum* acts as a middle colonizer—a bridge between early and late colonizers^59^—and facilitates the adhesion of early colonizer *Streptococcus sp.* via an adhesion protein called RadD.^60^ Simultaneously, *F. nucleatum* provides its serotype and lecitin-carbohydrate-specific adhesins to the late colonizers—*P. ginigvalis*, *A. actinomycetemcomitans* and *Treponema denticola*.^61, 62^ Similar to early colonizers, the late colonizers also show co-adherence among themselves. For example, *T. denticola* secretes chymotrypsin-like proteinases that aid in adhering to the existing late colonizers in order to form polymicrobial community.^63^ Further, succinate formed by *T. denticola* is exploited by *P. ginigvalis*, which in turn promotes the growth of *T. denticola* by providing isobutyric acid^52^ (Fig. 6).

In addition to the above-listed resident colonizers, both commensal and pathogenic bacteria have been shown to form biofilm structures. For instance, *P. intermedia* is a commensal to the healthy gingival crevices,^64^ while *P. gingivalis* is responsible for its invasion, resulting in the periodontal disease.^65^ Once within the gingival crevices, *P. gingivalis* aids *P. intermedia* to form biofilm structures with the help of virulence factors like arginine (Rgp) and lysine-specific cysteine proteases (Kgp).^66^ Simultaneously, *Porphyromonas ginigvalis* acts to detach *A. actinomycetemcomitans* from within the biofilms with the help of Kgp.^67^ Thus, bacteria can invade healthy gingival crevices by detaching and distorting the already existing oral biofilm. This can damage connective tissue, periodontal ligament, and bone with the help of bacterially secreted peptides and proteases, thereby allowing the bug access to bloodstream. Upon gaining entry into the coronary vasculature, these migratory bacteria can form biofilm structures within atherosclerotic plaques. For example, *F. nucleatum* and *Streptococcus sp.* forms a corncorb-like structure within the human atherosclerotic plaque.^68, 69^ Thus, these atherosclerotic plaque-associated bacteria may form mutually beneficial poly-microbial communities.^70^

## Discussion

The oral cavity is a complex part of the human system and a number of factors work in synergy to maintain its homoeostasis. The oral system serves as a major route for the entry of bacteria to populate and establish a microenvironment within the human system. In this respect, the oral commensal bacterial species not only maintain harmony within themselves via formation of biofilms and polymicrobial communities, but also with the host body by educating the immune system and contributing toward health.^71, 72^ The compositions of saliva and commensal bacterial populations in the oral cavity are inter-related, and over 700 bacterial species are housed.^71, 72^ For instance, the host glycoprotein component of saliva provides nutrition to the oral bacteria, whereas antimicrobials peptides secreted by the host system present in saliva keep the oral microbial populations in-check.^73^ Alternatively, the oral bacteria secrete proteins that degrade host defense peptides (anti-microbial) in saliva to sustain in the oral cavity.^74, 75^ Hence, the oral microbiome is increasingly considered a very significant player in human health and disease. In this study, we have shown the potential of the 23 atherosclerotic plaque-associated oral commensal bacteria in disease pathology. The cohort of 23 atherosclerotic plaque-associated bacteria is dominated by gram-negative bacteria with the exception of *Streptococcus sp*. Full genomes of 19 of the 23 bacteria from this cohort are now available, except *E. corrodens*, *E. hormaechei*, *F. necrophorum*, and *P. nigrescens*. Hence, a genomic platform has been established to enable bacterial and molecular profiling of factors that contribute to plaque formation. Further investigations of these microbe–plaque axes are now required to unravel the full extent of linkage between host microbiome with atherosclerosis.

## Methods

### Data collection

**Fig. 7:**
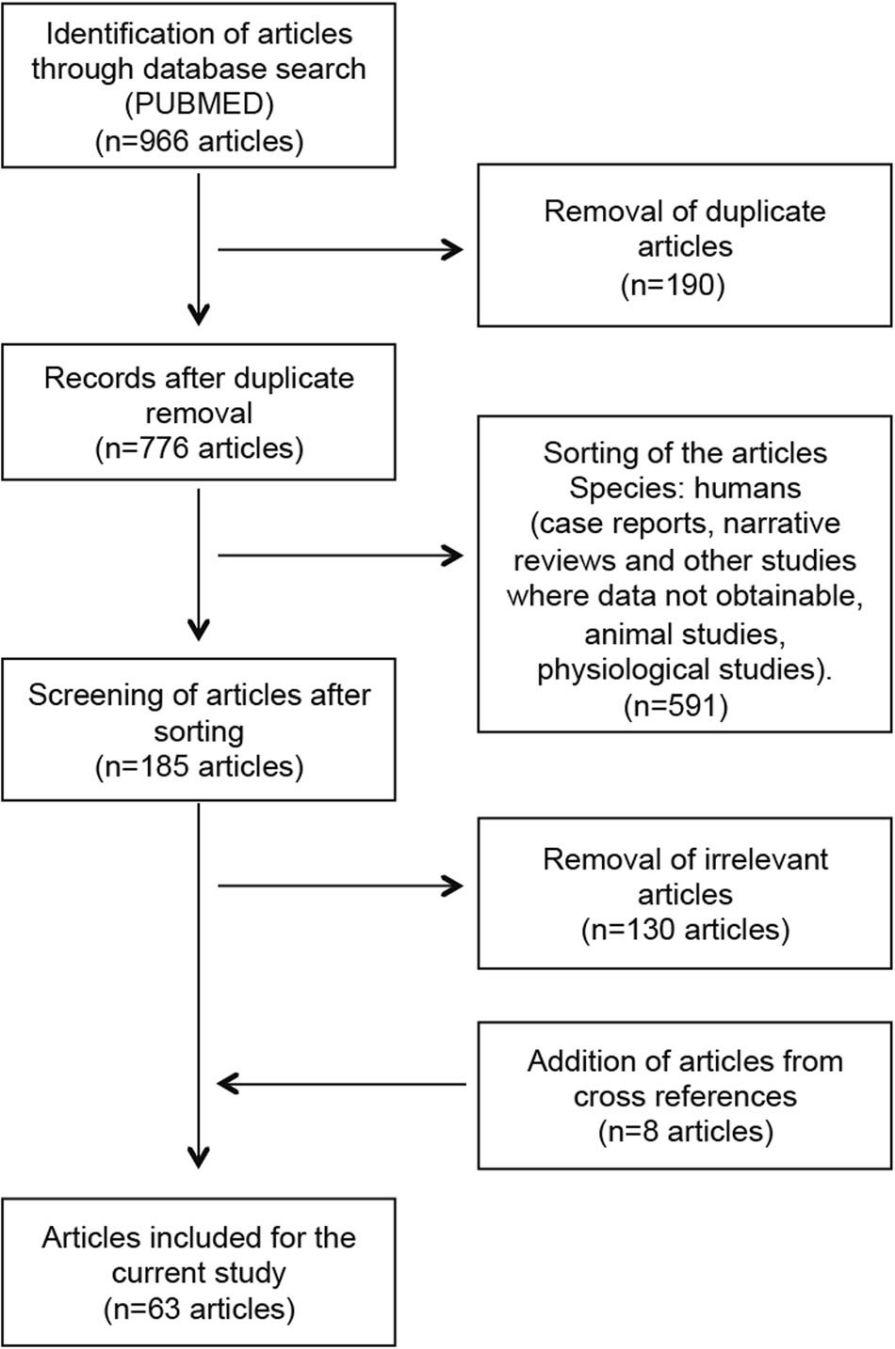
Study selection criteria to determine the population of microbes present in atherosclerotic plaques of CAD patients. Cataloguing procedure used to annotate all known oral microbes that have been identified from atherosclerotic plaque samples of CAD patients. In brief, the records present in PUBMED were studied by using combination of terminologies such as microbes, microorganism, and bacteria along with CAD, cardiovascular disease, atheroma, atherosclerosis, and atherosclerotic plaque. The articles were collated, duplicates removed, and relevant data extracted

Data sets selected in this study were sourced from published material from PUBMED, ATCC, and online web sources (Kenyon Microbe Wiki and Google search). Using a cataloguing procedure described in Fig. 7, we annotated all known bacteria that have been identified from atherosclerotic plaque samples of CAD patients. In brief, all PUBMED titles and abstracts were screened for eligibility. Any pre-clinical study was eligible for inclusion if it reported data regarding the presence of certain bacteria in atherosclerotic plaque within a coronary artery. These studies included randomized controlled trials, prospective case series, and controlled studies. Single case reports, conference proceedings, abstracts, and letters to the editor were screened but excluded if essential methodological information was missing. Additional articles from cross-references, which were missed due to absence of the search terms in title or abstracts, were hence included. Our literature search therefore resulted in selection of 63 studies, and identification of 23 bacteria in the human atherosclerotic plaque samples.

### Characterization on the basis of tissue localization and disease caused

Data sets selected in this study were sourced from published material from PUBMED, ATCC, and online web sources (Kenyon Microbe Wiki and Google search). In brief, all PUBMED titles and abstracts were screened for tissue localizations and diseases caused by the 23 atherosclerotic plaque-associated bacteria. Using above data, a map showing the presence of bacteria in various organ systems was generated. A GG plot of bacteria and diseases caused by them was prepared in R programming language.

### Identification of secretory proteins

The data pertinent in cataloguing secretory proteins/peptides from the 23 atherosclerotic plaque-associated bacteria were sourced from PUBMED. The screening process included only those studies that incorporated proper characterization of proteins/peptides secreted by any of the microbes in focus here. This resulted in a total of 33 articles whose data were added to this study.
